# Doctors

**Published:** 1873-04

**Authors:** 


### DOCTORS.

DR. NED.

In this number of the Bistoury I promised to say a few words about Doctors, but I am almost inclined to abandon the project for fear that I may not do the subject justice, and get "hauled over the coals" by some pill-peddler anxious for a controversy, or be made a matter of remark by some spicy editor, who thinks that everybody can be funny always because he can. I do not yet know the "style" of this article, it may get to be serious, savage or comic.—Actors generally perform for their audience, writers for their readers, philosophers for their subjects, and clowns for their pay. Doctors as a class are a strange set of men.—Excuse me, if you please, they used to be men, in the good old times of our fathers, when life was a grand and sublime reality instead of a show, swindle and farce. But now-a-days, the matter is somewhat mixed, and we must designate the class as a set of persons of the male and female persuasion. Women in the dark ages were advised by such stupid fellows as St. Paul, to be a little modest, keep house well, make it as comfortable, as circumstances would allow, for their husbands, have good coffee with sweet cream, and light buck-wheat cakes for breakfast; wash and dress the children, and train them for immortality, and do all things in that line according to the best of their judgment; but now, alas! how changed! women want to vote, smoke, cut wood, wear the best water proof boots, stand-up-collar, rowdy hat, spike-tail-coat, and practice medicine or law, or preach or swear, just like the rest of the fellows, while their poor, feeble husbands are at home, with a check apron on, rocking the babies or sweating at the washtub. So we see that things are getting down to a proper and correct level. I always knew that I would sooner crimp cap borders, mix bread, and raise a family, than to cut wood, pitch hay, or work in the rain, to support a big, stout, able-bodied, two fisted woman, competent to devour me. The fact is, nature made a big blunder at the send off, and we have just found it out. We are now getting into a delectable age, and I for one am willing to say with all good, pious, well meaning "boys," go it, Hannah Mary, I'll hold your bonnet." (Please don't make a mistake and print *bustle*, for bonnet, or the men folks will be discouraged at the size of the load they are to hold, and I would look as ridiculous as the boy with a picture of the world on his head, to be seen all alone, supporting such an intellectual monstrosity as a modern bustle, without its feminine attachment). I started out to write about doctors, and you must pardon this digression, and, beside, I trust that it will not be understood that I am fighting the women, for that would be too mean for anything; bless their sweet little souls, if they want to work for once, do not let any one attempt to stop them. I never knew but one man contemptable enough to make the effort, and he was a doctor who used to hire his own children for two cents to go to bed without their supper, and then make them pay it back for their breakfast. Of course that gentleman got rich. I will tell you a few words about him: He professed to be a saint, and of all men I ever knew he was the most jealous of his profession; would spurn everything and everybody who did not travel the beaten track, exactly as it was marked out by the society to which he chanced to belong. He was always ready and willing to abuse any man or woman who would not bow the neck for their professional yoke, or allow him to put thereon the collar and mark it "my dog." He pretended to be in love with his profession, but exhibited his ignorance of it by his hatred of every person who chanced to find favor among the people, or who pretended to deny that he knew everything worth knowing. Of all men I ever saw, he was the most perfect illustration of that remarkable text, "the fool is wise in his own conceit." Why, I knew of a gentleman who met this man in consultation once, and afterwards was employed professionally by some of his friends, which so enraged him that he absolutely went about telling the stupendous lie that he had taught the innovater all he knew, and although he pretended to be a saint, he would abuse the gentleman every time he met him, and would not have anything to do with him, or with any person, society, board of trade, or anything else, that would countenance his fancied antagonist. It is too bad that such wise men should be so exclusive; they should recollect that they were born to die, and be glad to let their wisdom bless the world long after their forms are traceless in the dust. I met this wonderful man once myself; of course, I learned a great deal, how could I avoid it? The earth is illumined by the sun, and its cheering rays seem to laugh when nature smiles through her dew-drops and the rose and the ray kiss each other. How naughty it would be for the big sun to jump up and down, kick, flounce, rave, roar and paw in madness, because he had lighted a world, when he was as brilliant as ever! How egotistical it would be for him to go about to all the planets, boasting of his doings, and abuse the little stars that glitter with joy, while the mellow moonbeams were bathing nature in her repose, and yell, scream, and insult the moon because she was not all light, like himself; proceedings like these would be superlatively ridiculous! Great, ponderous, gigantic intellects, should be willing to allow the smaller ones just to rub against them for a minute, it makes them so keen and happy.

I notice that large, clear, pure lakes, able to bear upon their bosoms the commerce and interests of a nation, permit small fish to romp and play as they please, beneath the surface, never heeding the commotion; whilst the shallow little pool is riled by the stir of a single tad-pole. When I see doctors assume to know everything and act mean and unmanly toward others, I always know that some tiny tad, too big for the capacity of the brook, has wiggled. This man, who had become so arrogant, and who had for so many years pretended to be the very centre of Christianity and wisdom, had at last come to the conclusion that it was true, just as I have heard of persons telling a falsehood so often that they believed it themselves. Why, it was no very uncommon thing for him to declare that he could not procure many medicines he desired in America, but would send to Paris, London, Liverpool, or Jerusalem, for compounds, and sell bottles of patent medicines, bought at a drug store, for eighty cents, to some of his poor christian sisters for twelve or fourteen dollars; declaring them to be importations from the Holy Land or the Celestial Empire. There seems to be something about the medical profession that contracts the mind and shrivels up the souls of men, instead of standing them upon a broad platform and giving to them enlarged and progressive views; and probably one of the most potent reasons why this is so, arises from the exclusive character of Medical Societies, which are so organized that their members are tied hand and foot, and are so depraved by it that they at last luxuriate in bondage like the unfortunate African who rejoices in his claims that he was a "high priced nigger." Business men form associations, and listen with interest to other's methods, and listening learn, and learning act. Clergymen, lawyers, everybody, seem anxious to push ahead and to embrace every opportunity, or call into requisition any agent calculated to advance their calling; but the poor, unfortunate, manacled doctor, if he chances to look out into the world, or desires in secret to wander a while in the realms of literature or science, not tread by his associates, he only looks like the hungry horse gazing upon the green fields beyond, but doomed to stay and starve in the barren waste around him, without the disposition or the power to break the cruel fetters he has worn so long.

Harvey broke the cords that bound him, and far in advance of the profession, he stood with his discovery of the circulation of the blood, on the mountains of fame for twenty years, while professional whiffets were barking at its base. He chose rather to benefit the race and immortalize himself, than to be a pharisee or wear a poke. Jenner, by his advance, made himself the target for the envious and the slave, but no matter to him, for he looked upon the intellect and the soul as too sacred and too mighty to be chained. The people themselves are far ahead of the lame and sore members of the profession who have been reposing upon iron bedsteads, and with feelings of pity they look back upon the unfortunate, and with one accord exclaim, "men, tear off your collars, do good, be happy and free."

